# Supplementary material for: Crosstalk Between Ethylene and Abscisic Acid During Changes in Soil Water Content Reveals a New Role for 1-Aminocyclopropane-1- Carboxylate in Coffee Anthesis Regulation
Source: Front Plant Sci. 2022 Apr 6;13:824948. doi: 10.3389/fpls.2022.824948 (PMC9019592; doi:10.3389/fpls.2022.824948)
Supplement: Supplementary file 1 [file Presentation_1.pdf]

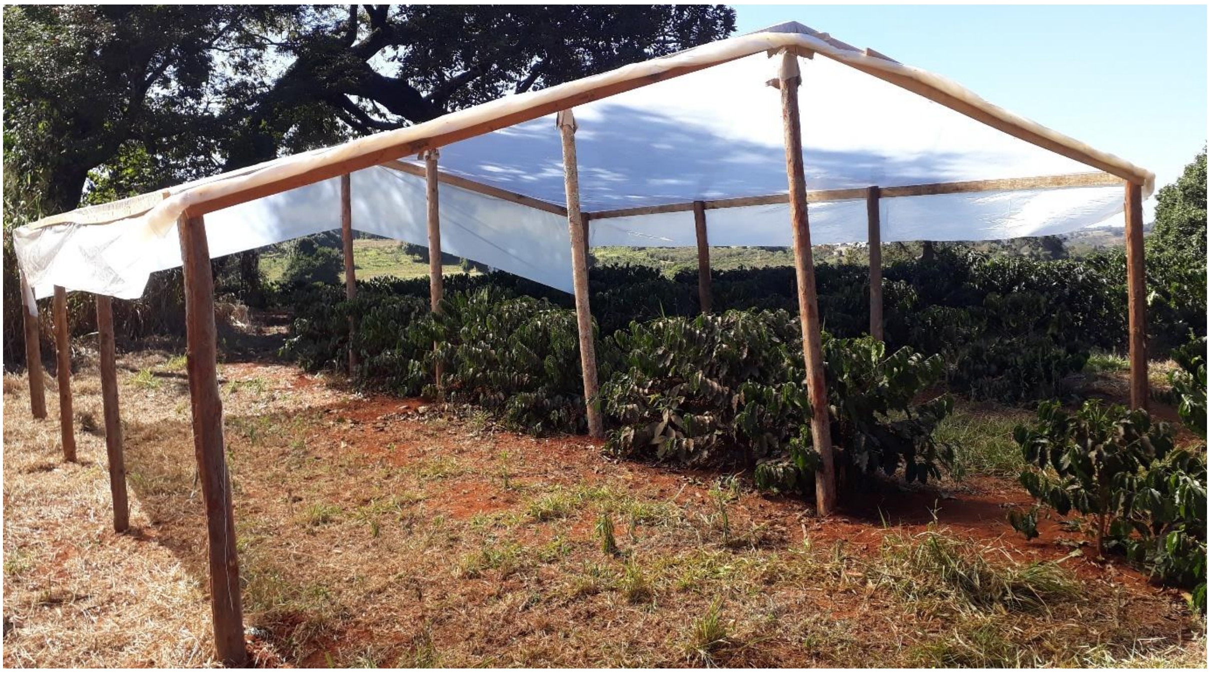

Supplementary Figure 1. Design of rainfall exclusion treatment in field experiment II. Rainfall exclusion was achieved through the installation of translucent nylon (polypropylene) fixed on twelve 3.2 m high eucalyptus wood logs, buried 0.60 m deep in the soil with three wood logs 3 m distant from each other. Every eucalyptus wood log was arranged on each posterior side and in the middle of the coffee line. A translucent nylon piece of 0,2 micrometers was fixed on top of wood logs using a wooden piece of 5x7 cm with small hobnail, covering a total of 81 m^2 of rain exclusion area

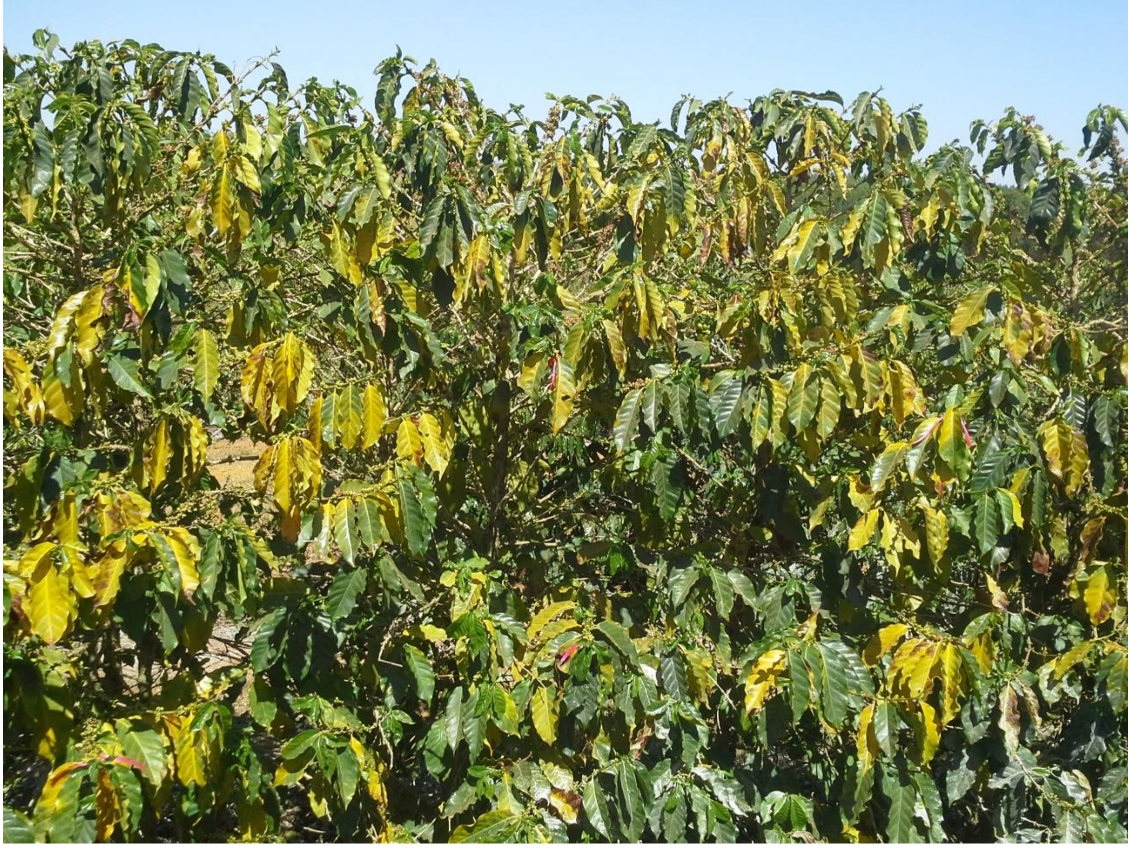

Supplementary Figure 2. Effect of ethylene application in coffee plants. All concentrations tested accelerated the leaf senescence process and promoted the abortion of flower buds

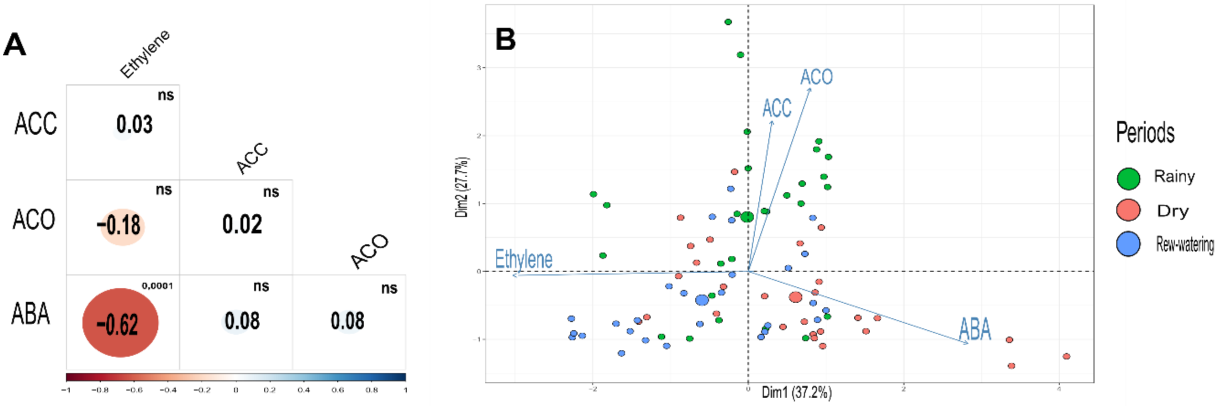

Supplementary Figure 3. Spearman Correlation **(A)** and PCA analysis **(B)** for ACC, ACO, ethylene, and ABA regulation in coffee anthesis. A principal component (PCA) and correlation analysis was carried out to understand the variation and relationship between the variables ABA, ACO, Ethylene, and ACC. The results showed that the biplot PCA represents 64,9% of the total variation. The main component 1 (37,2%) is the ethylene located in the positive part of the axis and by the ABA which is located in the negative part of the X-axis, which indicates that both have a negative correlation. The principal component 2 (27,7%) is represented by the variables ACO and ACC, which are located on the positive part of the Y-axis and are closely related to each other (Figure 3B). The results of the antagonistic relationship between ABA and ethylene are corroborated

by the Spearman correlation analysis where its coefficient is r = -0.62, showing a high negative correlation for the two variables

Table S1. Experimental design of the different experiments conducted and their specific hypotheses

Experiment	Treatments / Condition	Measurements	Hypothesis
Field I	Three coffee cultivars, Oeiras (Early), Acauã (Late) and *Semperflorens* (continuous) for coffee flowering pattern under field conditions in Rainy, Dry and Re-watering periods	-Predawn Leaf Water Potential<br>- ACC, ABA, Ethylene content, ACO activity and LHT1 gene expression	Hormonal crosstalk is involved in coffee anthesis
Field II	Rainfall exclusion, derived in Covered and Uncovered coffee plants in Re-watering period	-Predawn Leaf Water Potential<br>- ACC, ABA, Ethylene content, ACO activity and *LHT1* gene expression	Water is an important triggering transport signalling for coffee anthesis.
Field III	In coffee plants of *Semperflorens* were applied ACC, 1-MCP, (ACC+1-MCP) and Water treatments	-Flower bud in G6 stage	Exogenous application of ACC modulates coffee anthesis

Table S2. Sequence of primers used for RT-qPCR, where: TM = the melting temperature and E = efficiency of the primer annealing

Gene	Sequence of primers (5' – 3')	Concentration (µM)	Volume (µL)	TM (°C)	E (%)	Reference
Lysine histidine transporter (LHT1)	F: TTCGTCGGTTGCTCATCTCA<br>R: TTGCCTTCTCTTCAGCCGTT	1,5	2,25<br>2,25	54<br>52	100	This study
Malate dehydrogenase (MDH)	F: CCTGATGTCAACCACGCAACT<br>R: GTGGTTATGAACTCTCCATTCAACC	2	3<br>3	59<br>60	87	De Carvalho et al (2013)
Large ribosomal subunit 39 (RPL39)	F: GCGAAGAAGCAGAGGCAGAA<br>R: TTGGCATTGTAGCGGATGGT	2	3<br>3	59<br>60	87	Fernandes-Brum et al (2017)

Table S3. Quantitative real-time PCR parameters according to the Minimum Information for publication of Quantitative real-time PCR Experiments (MIQE) guidelines derived from Bustin *et al*. (2009)

**Experimental design / Sample**	
Experimental group	Four- and five-years old coffee (*Coffea arabica*) plants (Field experiment I, II) respectively.
Sample	Leaves, Roots and flower buds for both experiments

Sampling procedure	Immediately frozen in liquid nitrogen (roots were first washed and then dried using paper towels)
Storage conditions / Time of storage	Freezer -80 °C / 3 weeks
**RNA extraction**	
Processing procedure	Grinding (mortar and pestle) in liquid nitrogen
Method	Organic extraction, according to De Oliveira et al., 2015.
RNA: DNA-free	TURBO DNA-*free*™ Kit (Ambion, Thermo Fisher Scientific)- Catalog number: AM1907 - Design of intron-spanning primers whenever possible
Nucleic acid quantification	Spectroscopy (NanoVue GE Healthcare)
RNA integrity	Agarose gel (1 %)
**Reverse transcription**	
Kit	High-Capacity cDNA Reverse Transcription Kit (Applied Biosystems, Thermo Fisher Scientific) - Catalog number: 4368814
Reaction conditions	25 °C 10' / 37 °C 120' / 85 °C 5'
Reverse transcriptase	MultiScrib™ MuLV
Amount of RNA / Reaction volume	1 µg / 20 µL
Priming strategy	Random primers
Storage conditions of cDNA	Freezer -20 °C
**RT-qPCR**	
Target information	Table S2
Reaction conditions	As stated in the Materials and Methods section
In silico	Primers were blasted using the BLAST tool at https://www.ncbi.nlm.nih.gov/
Empirical	Primer concentration of 1µM (final concentration on the reaction)<br>Annealing temperature: 60 °C
PCR efficiency	5-fold dilution series of a mixed sample over at least five dilution points and verified to be higher than 80% (E = 10−1/slope).
Linear dynamic range	Samples are situated within the range of the efficiency curves for each primer
No template control (NTC)	Cq and dissociation curve verification
**Data analysis**	
Specialist software	Qiagen Rotor Gene-Q Series software (version 1.7)
Normalization	Two reference genes / $\Delta\Delta C_T$ method (Pfaffl, 2001)
